# Seom guidelines for the treatment of gastric cancer 2015

**DOI:** 10.1007/s12094-015-1456-y

**Published:** 2015-12-21

**Authors:** M. Martin-Richard, A. Custodio, C. García-Girón, C. Grávalos, C. Gomez, P. Jimenez-Fonseca, J. L. Manzano, C. Pericay, F. Rivera, A. Carrato

**Affiliations:** Medical Oncology Department, Hospital de la Santa Creu I Sant Pau, 167, 08025 Barcelona, Spain; Medical Oncology Department, Hospital Universitario Clínico San Carlos, Madrid, Spain; Medical Oncology Department, Hospital Universitario de Burgos, Burgos, Spain; Medical Oncology Department, Hospital Universitario 12 de Octubre, Madrid, Spain; Medical Oncology Department, Hospital Universitario Central de Asturias (HUCA), Oviedo, Spain; Medical Oncology Department, Hospital Universitari Germans Trias I Pujol de Badalona, Barcelona, Spain; Medical Oncology Department, Hospital de Sabadell-Consorcio Sanitario Parc Taulì, Barcelona, Spain; Medical Oncology Department, Hospital Universitario Marqués de Valdecilla, Santander, Spain; Medical Oncology Department, Hospital Universitario Ramón y Cajal, Madrid, Spain

**Keywords:** Gastric adenocarcinoma, Cardia, Diagnosis, Treatment, Clinical guidelines

## Abstract

Gastric cancer is the fourth cause of death by cancer in Spain and a significant medical problem. Molecular biology results evidence that gastroesophageal junction tumors and gastric cancer should be considered as two independent entities with a different prognosis and treatment approach. Endoscopic resection in very early tumors is feasible. Neoadjuvant and adjuvant therapy in locally advanced resectable tumor increase overall survival and should be considered standard treatments. In stage IV tumors, platinum–fluoropyrimidine-based schedule, with trastuzumab in HER2-overexpressed tumors, is the first-line treatment. Different therapies in second line have demonstrated in randomized studies their clear benefit in survival improvement.

## Introduction

Gastric cancer (GC) is a major contributor to the global cancer burden. In 2012, there were an estimated 951,000 new cases worldwide, 6.8 % of the total, making GC the fifth most common malignancy [1]. GC is the third leading cause of cancer death in both sexes worldwide with 723,000 deaths, 8.8 % of the total. In Spain, GC was the sixth most common malignancy, with 7810 new cases and 5389 deaths [2]. Geographically, almost two-thirds occur in Asia, with 43 % of total global cases in China. Incidence rates are low in developed regions such as North America and Western Europe. GC is more frequent among males than in females (11.0 versus 5.1 per 100,000) and its incidence increases with age (peak presentation is between 65- and 74-year old). Mortality age-standardized rate is 9.7 in men and 4.6 in women [3]. The most important familial cancer syndrome is hereditary diffuse gastric cancer syndrome [mutations in the germline E-cadherin mutations (CDH1) gene] [1].

Although often reported as a single entity, GC can generally be classified [4] into two topographical categories: (1) cardia gastric cancer (CGC) arising in the area of the stomach adjoining the esophageal–gastric junction [5], and (2) non-CGC (NCGC) arising from more distal regions of the stomach. Risk factors for CGC include obesity, gastro-esophagus reflux disease (GORD) and Barrett’s esophagus. NCGC, however, is strongly associated with Helicobacter pylori infection, socioeconomic status (SES), excess nitrates, and atrophic gastritis. Both types are influenced by cigarette smoking and by low intake of fruits and vegetables. Given these differences, there is increasing interest in describing the worldwide burden of CGC and NCGC subsites separately [6]. CGC: there were 260,000 new cases, comprising 27 % of total gastric cancer worldwide (30 % in men and 21 % in women). Age-standardized rate was 3.3 per 100,000 (5.3 versus 1.6 in men and women, respectively). NCGC: there were 691,000 new cases, 70 % in men and 79 % in women. Age-standardized rate was 8.8 per 100,000 (12.1 versus 5.9 in men and women, respectively). In most countries, CGC incidence rates are lower than NCGC rates (91 % of 184 GLOBOCAN countries).

These clinical guidelines aim to offer succinct, practical recommendations for diagnosis, treatment, and follow-up of GC.

## Molecular classification of gastric cancer

Gastric cancer is known as a heterogeneous disease, which may be divided into subgroups based on histological, anatomical, epidemiological, and also molecular classifications [7–12].

Despite such incredible advance in our knowledge of gastric cancer genomics, there is little overlap between the published classifications, and the reproducibility of the different studies is very poor [13]. Therefore, it is difficult to compare expression data from different studies and such inconsistency limits our ability to develop robust and reliable molecular models.

## Molecular prognosis of gastric cancer

There is no one single molecular alteration that has been universally accepted as independent prognostic factor in gastric cancer. Many gene expression signatures have been able to classify tumors into intrinsic subtypes and predict the survival of GC patients. Some studies have showed that gene expression profiling can predict patients with a high risk for recurrence after curative surgery [14–16]. Other studies using gene expression data with proper clinical information have developed predictive models that could identify long survivors, either by identifying specific gene signatures that correlated with the overall survival [17–19] or by identification of Genomic signatures that could successfully predict the relapse of GC, specially in form of peritoneal relapse [20].

Despite all these profits in the development of prognostic and predictive molecular models for GC, they are at an early stage and in need of substantial improvement to be ready for its clinical implementation.

## Diagnosis and staging

### Diagnosis

Gastroscopy and biopsy of suspicious lesions are the basis for definitive diagnosis. Histology is reported according to World Health Organization criteria [21] and Lauren classification (intestinal and diffuse). Immunohistochemistry (IHC) determines HER2 overexpression in advanced disease, according to GC-specific criteria [22], to decide trastuzumab treatment.

### Staging

WHO performance status (PS), nutritional status, and comprehensive geriatric assessment in the elderly, as well as physical exam, liver, renal and blood tests, and tumor stage should be considered before choosing treatment;

Locoregional disease:

- Endoscopic ultrasound (EUS) and ultrasound-guided fine-needle aspiration of suspicious lymph nodes inform about locoregional disease spread and are optimal to distinguish T2–4 staging [IIA] [23] or
- Esophagus-gastro-duodenal transit, when endoscopy cannot be performed.

Distant disease:

- Computed tomography (CT) is standard to confirm metastases (IA).
- Laparoscopy, peritoneal washings, and cytology is mandatory in locally advanced gastric (T3–4 and/or node-positive disease) and esophagogastric junction cancer [IIA] [24]
- The value of integrated PET/CT in patients who are offered curative surgery is a subject to debate, although it may be convenient in large tumor size, non-signet ring cell, non-mucinous, non-diffuse carcinoma type, and glucose transporter 1-positive expression on immunohistochemistry [25].
- Combinations of CEA, CA19-9, and CA72-4 are the most effective serum tumor markers for staging, detection of recurrence, or evaluation of the response [26].
- Staging is performed according to the 2010 AJCC TNM classification, 7th edition (Table 1) [4]. Four major groups are considered for clinical management purposes (Table 2).

**Table 1:** Tumor stage of gastric cancer according to AJCC 2010

Tx	Primary tumor cannot be assessed		
T0	No evidence of primary tumor		
Tis	Carcinoma in situ: intraepithelial tumor without invasion of the lamina propria		
T1	Tumor invades lamina propria, muscularis mucosae, or submucosa		
T1a	Tumor invades lamina propria or muscularis mucosae		
T1b	Tumor invades submucosa		
T2	Tumor invades muscularis propria^a^		
T3	Tumor penetrates subserosal connective tissue without invasion of visceral peritoneum or adjacent structures^b^		
T4	Tumor invades serosa (visceral peritoneum) or adjacent structures^b^		
T4a	Tumor invades serosa (visceral peritoneum)		
T4b	Tumor invades adjacent structures		
Nx	Regional lymph node(s) cannot be assessed		
N0	No regional lymph node metastasis^c^		
N1	Metastasis in 1–2 regional lymph nodes		
N2	Metastasis in 3–6 regional lymph nodes		
N3	Metastasis in seven or more regional lymph nodes		
N3a	Metastasis in 7–15 regional lymph nodes		
N3b	Metastasis in 16 or more regional lymph nodes		
Mx	Distant metastasis cannot be assessed		
M0	No distant metastasis		
M1	Distant metastasis		

Stage	T	N	M
Anatomic stage/prognostic groups			
0	Tis	N0	M0
IA	T1	N0	M0
IB	T2	N0	M0
	T1	N1	M0
IIA	T3	N0	M0
	T2	N1	M0
	T1	N2	M0
IIB	T4a	N0	M0
	T3	N1	M0
	T2	N2	M0
	T1	N3	M0
IIIA	T4a	N1	M0
	T3	N2	M0
	T2	N3	M0
IIIB	T4b	N0	M0
	T4b	N1	M0
	T4a	N2	M0
	T3	N3	M0
IIIC	T4b	N2	M0
	T4b	N3	M0
	T4a	N3	M0
IV	Any T	Any N	M1

cTNM is the clinical classification, pTNM is the pathologic classification

Primary tumor (T), Regional lymph nodes (N)

^a^A tumor may penetrate the muscularis propria with extension into the gastrocolic or gastrohepatic ligaments, or into the greater or lesser omentum, without perforation of the visceral peritoneum covering these structures. In this case, the tumor is classified T3. If there is perforation of the visceral peritoneum covering the gastric ligaments or the omentum, the tumor should be classified T4

^b^The adjacent structures of the stomach include the spleen, transverse colon, liver, diaphragm, pancreas, abdominal wall, adrenal gland, kidney, small intestine, and retroperitoneum. Intramural extension to the duodenum or esophagus is classified by the depth of the greatest invasion in any of these sites, including the stomach

^c^A designation of pN0 should be used if all examined lymph nodes are negative, regardless of the total number removed and examined

**Table 2:** Prognosis and treatment options

Groups	Early resectable disease (10 %)	Locally advanced resectable disease	Locally advanced unresectable disease (20 %)	Metastatic disease (30 %)
Stages	Stages 0–I;	II–IIIC	Some IIIB–IIIC	IV
5-year/median OS	70 %	30–40 %	12–14 months	9–11 m with CT 4 m without CT
Treatment	Surgery or Endoscopic resection	Perioperative, Neoadjuvant o Adjuvant ttm.	CT	CT

*CT* chemotherapy, *ttm* treatment

## Treatment

### Early gastric cancer

#### Endoscopic resection

Early GC (T1a) may be amenable to endoscopic resection if it is well differentiated, <2 cm, confined to the mucosa, and non-ulcerated. Intestinal Lauren histology and no evidence of lympho-vascular invasion also indicate mucosectomy in: intramucosal cancers without ulceration, regardless of tumor size; ulcerated intramucosal cancers <3 cm, or cancers with early invasion into the submucosa measuring <3 cm, endoscopic submucosal dissection has proven more effective than endoscopic mucosal resection, but requires greater skills and instrumentation and entails significant risk of complications, including perforation [27]. The risk of lymph node metastases following endoscopic resection by experts remains low in these tumors [28]. In less experienced centers, limited surgery is an alternative. T1A GC not meeting criteria for endoscopic treatment will require less extensive surgery than IB-III tumors and lymph node dissection can be limited to peri-gastric and local nodes (Table 3).

**Table 3:** Treatment recommendations

Stage	Details	Treatment
Early stage: Tis		
T1a T1a T1b T1b	Well dif./<2 cm/non-ulcerated/intestinal Others non-ulcerated <3 cm Others	Endoscopic resection Endosc. resect mucosectomy/surgery Submucos. resect/surgery Surgery
Stage I		Surgery
Locally Advanced (Stage II–III)	Cardias GC	Neoadjuvant CT (IB)
		Or CRT (IA)
		Or Perioperative CT (IB)
		Or Adjuvant CT (IA)
		Or Adjuvant CRT (IB)
	Non-cardias GC	Perioperative CT (IB)
		Or Adjuvant CT (IA)
		Or Adjuvant CRT (IB)
Advanced disease (Stage IV)	First-line CT	
	HER2+	Cisplatin–Fluorop (IB)
	HER2 negative	PFluorop or EPFluorop (IA)
		Or TCF (IB)
		Or FOLFIRI or IF (IB)
	Second-line CT	Irinotecan (IA)
		Or Docetaxel (IA)
		Or Paclitaxel (IB)
		Or Ramucirumab (IB)
		Or Paclitaxel–Rramucirum (IB)

*Fluorop*. 5Fu or Capecitabine, *P* cisplatin or oxaliplatin, *TCF* taxotere + cisplatin + 5FU

### Locally advanced disease

#### Surgery

Complete resection with adequate margins remains the cornerstone of curative treatment. The type of resection in GC, subtotal versus total gastrectomy, depends on the anatomic location of primary tumor. For esophagogastric junction (EGJ) cancers, a total esophagectomy with a partial gastrectomy or an extended gastrectomy is generally performed.

Extent of lymph node dissection remains a subject of controversy. In eastern Asia, gastrectomy with D2 lymph node dissection is the standard treatment for curable GC. In Western countries, two large randomized trials failed to demonstrate a significant survival benefit for D2 over D1 lymph node dissection. However, mature 15-year follow-up data from the Dutch trial showed a lower locoregional recurrence rate and fewer GC-related death with D2 lymphadenectomy [29]. A recent meta-analysis of 12 randomized clinical trials confirmed no OS benefit for D2 lymphadenectomy, although a benefit was seen among patients who had resection without splenectomy and/or pancreatectomy [30]. There is uniform consensus that lymphadenectomy must include at least 15 lymph nodes. Gastrectomy with D2 lymph node dissection is a recommended procedure (2B), but should be performed by experienced surgeons in high-volume centers. Routine pancreatectomy and splenectomy are no longer recommended with D2 lymph node dissection.

#### Neoadjuvant treatment

##### Chemotherapy (CT)

A study by the EORTC (40954) in patients with locally advanced cancer of the stomach or EGJ found a significantly higher rate of R0 resection among patients receiving neoadjuvant CT; however, no statistically significant difference in survival was reached [31]. Some meta-analysis in GC has been conduced with conflicting results. A meta-analysis in EG cancers, where several EGJ were included [32], showed a survival benefit for neoadjuvant CT. Currently, we should consider neoadjuvant chemotherapy in gastroesophageal cancer (IB).

##### Chemoradiotherapy (CRT)

The phase III study POET compared preoperative CT with preoperative CRT in patients with locally advanced adenocarcinoma of the EGJ. Patients in the CRT group had a significant higher pathologic complete response, but statistical significance was not achieved for overall survival (OS) [33]. The phase III CROSS trial compared neoadjuvant CRT versus surgery alone in patients with squamous cell carcinoma and adenocarcinoma of the esophagus or EGJ, with a significant increase of OS in the neoadjuvant group [34]. The meta-analysis published by Sjoquist et al. [32] supports a survival benefit for neoadjuvant CRT compared to surgery alone for esophageal and EGJ cancer. Preoperative combined CRT is now the preferred approach for localized EGJ and gastric cardia cancers (IB). However, it is still an experimental procedure in potentially resectable non-cardia gastric adenocarcinomas (2B).

#### Perioperative treatment

The theoretical advantages of perioperative treatment are the potential increase of the complete resection (R0) rate, a better tolerability profile and increased probability of treatment compliance, early systemic treatment of micrometastatic disease, and an ideal scenario to assess the efficacy of scheduled treatment, as well as new agents.

Two phase III, adequately powered trials in Western countries, the MAGIC [35] and the FNLCC/FFCD 9703 [36], and some recent meta-analysis [37] have shown that perioperative chemotherapy (CT) significantly increases R0 rates, disease-free survival (DFS), and OS, and does not significantly increase perioperative complications or mortality with tolerable grade 3–4 toxicity rates (Table 1). These results have led to the adoption of perioperative CT as a standard approach for medically fit patients with resectable locally advanced (cT2 or higher, any N) distal esophageal, esophagogastric junction, or gastric tumors [IA] throughout most of European countries and other parts of the world.

Regarding the role of targeted agents in the perioperative setting, two phase II trials have tested the combination of trastuzumab and CT in HER-2-positive patients showing promising R0 rates and pathologic complete responses (pCR). However, the addition of bevacizumab to a perioperative regimen of epirubicin, cisplatin, and capecitabine (ECX) did not improve survival [38].

The potential benefit of adding postoperative or preoperative CRT to standard perioperative CT is being evaluated in two trials.

#### Adjuvant treatment

##### Chemoradiation

In patients with resected gastric or GEJ adenocarcinoma stages IB–IV (M0), the INT-0116 trial reported better OS (HR 1.35; *p* = 0.005) and DFS (HR 1.52; *p* < 0.001) with the MacDonald regimen (5FU/LV plus radiotherapy) versus surgery alone [39] (IB). In the 13-year follow-up, the benefit of adjuvant CRT was maintained and not substantial long-term toxicities were reported [40]. The CALGB 80101 compared the INT-0116 regimen with ECF (epirubicin, cisplatin, 5FU before and after 5FU/RT) in resected GEJ or gastric cancer without observing differences in 3-year OS (52 and 50 % for ECF and 5FU/LV, respectively). In HER2-positive tumors, the phase II TOXAG trial will analyze the safety of adjuvant oxaliplatin, capecitabine', and trastuzumab with radiotherapy.

##### Chemotherapy

The benefit of adjuvant CT has also been reported. An absolute increment of 6 % in OS (HR 0.82; *p* < 0.001) and a better DFS (HR 0.82; *p* < 0.001) were published in a large, individual patient-level meta-analysis of adjuvant 5FU-based chemotherapy versus surgery alone in resected GC [41] [I,A]. However, the preferred combination chemotherapy could not be determined.

In addition, the ACTS-GC randomized phase III trial showed a significantly better 3-year OS with S-1 for 1 year than with observation in D2-resection stage II or III GC patients [42] [IB]. In the update after 5 years of follow-up, the benefit was maintained in the S-1 group [43]. In the same type of patients, the XELOX regimen was superior to surgery alone in the CLASSIC phase III trial. With a median 5-year follow-up, the estimated 5-year DFS was 68 versus 53 % and the estimated 5-year OS was 78 versus 69 % in the XELOX and the surgery-only groups, respectively [44].

##### Adjuvant chemoradiotherapy vs chemotherapy

The ARTIST trial compared CRT [cisplatin and capecitabine (XP), with capecitabine and radiation concurrently] versus chemotherapy alone (XP × 6 cycles) in patients with at least D2 lymphadenectomy and R0 resection [45]. With a 7-year follow-up, the DFS (HR 0.74; *p* = 0.092) and the OS (HR 1.13; *p* = 0.527) were similar between the groups. Subgroup analyses showed that CRT significantly improved DFS in node-positive disease and with intestinal-type GC [46]. Finally, the Korean ARTIST II randomized phase III trial is currently comparing adjuvant S-1 versus S-1 plus oxaliplatin (SOX), with or without radiotherapy.

### Advanced disease

#### First-line treatment

Several randomized studies and a meta-analysis [47], comparing palliative CT with the best supportive care (BSC), have demonstrated that CT increases median overall survival and improves the quality of life of patients with advanced gastric cancer [I,A].

##### Cisplatin-based chemotherapy

In the late nineties, it was proven that regimes based on cisplatin were superior to other older regimes without cisplatin. Both CF (cisplatin–5FU) and ECF (epirubicin–cisplatin–5FU) can be considered standard combinations (IA). Although a meta-analysis suggests better OS with ECF, there are no randomized head-to-head comparison studies

##### Chemotherapy with docetaxel

In the Phase III study V-325 [48], docetaxel, cisplatin, and 5FU (DCF) was compared with CF. DCF was superior in TTP, OS, and response rate but was also more toxic. Triplets with Docetaxel can be considered in selected patients (IB).

##### Oxaliplatin-based chemotherapy

Two phase III studies [49, 50] have suggested that oxaliplatin has similar efficacy and less toxicity than cisplatin and can replace it in this setting (IA).

##### Oral fluoropyrimidines

Two phase III studies [49, 51] have observed no inferiority in efficacy and a more favorable toxic profile, when replacing 5FU with capecitabine. Another phase III study observed non-inferiority in efficacy and better convenience and toxicity with cisplatin-S1 versus CF [52]. Based on these data, oral fluoropyrimidines (capecitabine or S-1) can replace 5FU in this setting [I,A].

##### Irinotecan combinations

Irinotecan/5FU/LV combinations (FOLFIRI, IF) have been compared with CF in two randomized trials [53, 54] showing similar efficacy and better tolerance and can be considered adequate options for these patients (I,B).

##### Trastuzumab

The phase III TOGA [55] analyzed HER-2 status in 3807 patients. Twenty-two per cent were HER-2+ (594) and were randomized to receive chemotherapy alone (CX or CF) or chemo plus trastuzumab. In the trastuzumab arm, it was shown a statistically significant increase in the primary endpoint, which was OS as well as in PFS. A preplanned analysis of OS in the subgroup of patients with IHC 3+ or IHC 2+ with FISH+ showed an increase of 5 months OS in the trastuzumab arm (16.8 versus 11.8 months, HR 0.65). According to these results, we can consider that trastuzumab added to cisplatin + fluoropyrimidines can be considered the standard treatment in patients with gastric or EGJ advanced HER-2+ adenocarcinoma [IB].

#### Second-line chemotherapy

Less than 60 % of patients receive second- or third-line therapy for gastric cancer in clinical practice [55]. There is evidence that second-line chemotherapy achieves improvement in quality of life (QOL) and provides a median overall survival (OS) of 4–6 months [I,A]. Docetaxel, paclitaxel, irinotecan, and a targeted therapy against vascular endothelial grow factor receptor 2 (VEGFR2) with ramucirumab have demonstrated a significant benefit in OS in phase III trials [I,B]. Two recent meta-analysis have shown a significant reduction in the risk of death [56, 57] [I,A]. The reduction was HR = 0.55 for irinotecan, and HR = 0.71 for docetaxel [57].

*Irinotecan* The first study randomized 40 patients between irinotecan and best supportive care (BSC). Irinotecan showed a statistically significant survival benefit over BSC (median OS 4 versus 2.4 months, respectively) [I,B] [58].

A Korean trial randomized patients to BSC, to 3-weekly docetaxel or 2-weekly irinotecan. Chemotherapy showed a significant survival benefit (median OS 5.3 versus 3.8 months, HR = 0.65) over BSC alone [I,B] [59].

*Docetaxel* The UK COUGAR-02 trial randomized 168 patients to 3-weekly docetaxel versus BSC. Docetaxel improved median overall survival over BSC (5.2 months versus 3.6 months, respectively, HR = 0.67) [I,B]. Global quality of life (QOL) scores were similar between the two arms [60].

*Paclitaxel* A phase III trial with 219 Asian patients did not show a superiority of irinotecan against paclitaxel [I,B] (median OS 8.4 versus 9.5 months, respectively, HR = 1.14) [61].

Ramucirumab has shown a statistically significant efficacy as monotherapy (REGARD) or in combination with paclitaxel (RAINBOW). REGARD randomized 445 patients to ramucirumab versus placebo. Ramucirumab showed a significant OS benefit (5.2 months versus 3.8 months, HR = 0.77) over placebo [I,B] [62].

RAINBOW randomized 665 patients to ramucirumab plus paclitaxel or to paclitaxel plus placebo. Ramucirumab plus paclitaxel arm showed a significantly superior OS (9.6 months versus 7.3 months, HR = 0.80) over paclitaxel monotherapy [I,B] [63].

### Future lines and targeted drugs

Since the approval of Trastuzumab in advanced HER2 positive gastric cancer, in 2010, there have been an increasing number of molecules and targets entering the preclinical and clinical research programs in GC.

New strategies underway to improve the results of trastuzumab in HER2-positive GC include the use of antibody–drug conjugates to deliver cytotoxic agents such trastuzumab emtansine (T-DM1) [64] or its combination with new monoclonal antibodies (MoAbs) that targets a different extracellular dimerization domain (Pertuzumab) [65]. Antiangiogenic therapy with Ramucirumab, a fully human immunoglobulin (Ig) G1 monoclonal antibody targeting VEGFR-2, has demonstrated improved survival both as monotherapy and in combination with paclitaxel [62, 63] in the second-line setting. By the contrary, 2 MoAbs targeting MET were unsuccessfully tested in this disease [66, 67].

Two recently communicated clinical trials have demonstrated some early signs of activity of both antiPD-1 and antiPD-L1 antibody immune checkpoint inhibitors in upper GI malignancies [68, 69].

*Follow-up* In the setting of operable gastric cancer, a regular follow-up may allow treatment of symptoms and early detection of recurrence, though there is no evidence that it improves survival outcomes [III, B]. If relapse/disease progression is suspected, then physical examination, blood tests, and radiological investigations or endoscopy should be carried out. We recommend anamnesis and physical examination every 3–6 months in the first 3 years following surgical intervention and then every 6–12 months during years 4 and 5 [V]. The benefit of following up the patients beyond year 5 is controversial [70].
